# Long-Term Care Preferences and Sexual Orientation: Protocol for a Systematic Review

**DOI:** 10.3390/healthcare8040572

**Published:** 2020-12-18

**Authors:** Elżbieta Buczak-Stec, Hans-Helmut König, Lukas Feddern, André Hajek

**Affiliations:** Department of Health Economics and Health Services Research, Hamburg Center for Health Economics, University Medical Center Hamburg-Eppendorf, 20246 Hamburg, Germany; h.koenig@uke.de (H.-H.K.); feddernlukas@gmail.com (L.F.); a.hajek@uke.de (A.H.)

**Keywords:** sexual and gender minorities, long-term care, formal and informal care, sexual orientation, nursing home, LGBT, older people, ageing in place, systematic review, old age home, homosexuals, bisexuals

## Abstract

Background: With increasing age, the health status of older individuals commonly deteriorates and their care needs greatly increase. Therefore, many individuals are in need for formal or informal long-term care. In order to plan suitable long-term care settings, it is important to know the long-term care preferences of an ageing population (both heterosexuals and sexual minorities). The aim of this study is to systematically review the literature for evidence on preferences regarding long-term care and the potential differences with regard to sexual orientation. Methods and analysis: This study protocol for a systematic review is reported according to the PRISMA-P guidelines. A comprehensive search of published studies will be conducted using PubMed, Web of Science and PsycINFO bibliographic databases. Following predefined inclusion criteria, two authors will screen the titles and abstracts of the studies independently. Afterwards, we will obtain and screen full-text articles of eligible studies using the predefined inclusion criteria. Discrepancies will be resolved by consensus or consultation with a third researcher. Data will be extracted and synthesised. Extracted data will be categorised based on study design, type of long-term care preferences and the group (sexual orientation) which is addressed. The quality of reporting of the studies included will be assessed.

## 1. Introduction

In recent years, many initiatives have been conducted to promote healthy ageing and to enable ageing in place as long as it is possible [1,2]. On the other hand, many individuals are aware that with increasing age their health status commonly deteriorates, the care needs may greatly increase and at some point, living independently at home may be impossible [3]. For those reasons, providing proper care and proper accommodation for older individuals who cannot care for themselves is becoming an important issue for the health care system and for health policy. It important to identify the long-term care preferences of an ageing population. This knowledge is essential in order to plan suitable care, appropriate facilities, and long-term settings.

To date, various studies have examined preferences among older adults related to long-term care and moving into a nursing home (NH) in general [3,4,5,6,7]. Of these, some studies have examined long-term care preferences and preferences for NH care among Lesbian, Gay, Bisexual and Transgender (LGBT) people or sexual minorities [8,9,10]. Although older sexual minorities represent a large group within society [11,12,13], this group is not well researched. Some studies suggested that the preferences regarding long-term care may differ according to sexual orientation [14,15,16]. It has been suggested that older sexual minorities do not want to move into nursing homes as they fear discrimination and stigmatization due to their sexual orientation [17,18,19]. Contrarily, there is evidence showing no significant differences regarding the preferences between heterosexuals and sexual minorities [10]. Thus far, no study has systematically synthesized observational studies investigating the preferences for long-term care with regard to sexual orientation. Consequently, the goal of our upcoming systematic review is to close this gap in the literature.

The objective of this study is to present the protocol for a systematic review. The systematic review aims to answer the following questions: What are the preferences regarding long-term care among sexual and gender minorities (e.g., moving into a nursing home, informal or formal care provided at home, assisted living)? Moreover, we would like to know whether the preferences differ among heterosexuals and sexual minorities. This is an important issue for planning possible changes in the health care system or nursing home policy in order to correspond and adapt long-term care to the requirements and needs of every group.

## 2. Materials and Methods

The protocol was registered on the PROSPERO international prospective register of systematic reviews (registration number CRD42020202005). It is reported in line with the Preferred Reporting Items for Systematic Review and Meta-Analysis Protocols (PRISMA-P) guidelines [20].

### 2.1. Eligibility Criteria

Studies will be selected according to the criteria outlined below.

#### 2.1.1. Study Design

In our systematic review, we will include i.a. quantitative cross-sectional and longitudinal observational studies examining the association between sexual orientation and long-term care preferences, i.e., comparing gender and/or sexual minorities (e.g., homosexual vs. bisexual) and ideally (but not necessarily) heterosexuals (e.g., heterosexual vs. homosexual) with regard to long-term care preferences.

#### 2.1.2. Participants

In our systematic review, we will include studies examining the preferences for long-term care among community-dwelling individuals (18 years and older). We will exclude studies that exclusively focus on children or youths. Studies that are exclusively focusing on individuals living in institutionalized settings will also be excluded. In our research, we will include studies that focus either on both, sexual and gender minorities and heterosexuals, or that are including only sexual or gender minorities. Studies only including heterosexuals will be excluded. Studies exclusively focusing on preferences of nursing home staff will also be excluded.

#### 2.1.3. Outcome

Our outcome measure is the long-term care preferences, e.g., the preference to move into a nursing home in the future, preference for informal care or formal home care.

#### 2.1.4. Language and Publication Status

We will restrict our review to empirical publications in peer-reviewed, scientific journals. Moreover, selected articles are limited to those, written and published in English or German.

### 2.2. Information Sources

We will search the electronic databases (PubMed/MEDLINE, Ovid/PsycINFO, and Web of Science) using predefined search terms. The literature search strategy was developed based on medical subject headings (MeSH) and text words related to three terms: (i) nursing home, (ii) sexual and gender minorities and (iii) long-term care preferences. The reference lists of eligible studies and review articles will be searched manually (backward citation). Moreover, relevant cited articles will be analyzed (forward citation).

### 2.3. Search Strategy

The search strategy has been developed with substantive and methodological input from the project team. PROSPERO will be searched for ongoing or recently completed systematic reviews. Table 1 lays out the relevant search strings for sexual and gender minorities, long-term care, and preferences, respectively. As the initial strategy detailed in Table 1 was developed using PubMed-specific syntax, the search strategy is adapted syntactically for the other databases. The first 100 results of a pre-test confirmed the same application of selection criteria among researchers responsible for the screening.

### 2.4. Selection Process

Two reviewers will apply the specified eligibility criteria and will individually select studies for inclusion in the systematic review. They will independently screen records based on their title, abstract, and body for inclusion, respectively. That is, researchers will be blinded to each other’s decisions. Divergence (e.g., whether to include the study for the full text screening) of the reviewers’ judgements will be resolved through discussion. If the disagreements persist, a third reviewer will be consulted. We will use the following programs for a system or mechanism for recording decisions—MS Excel and EndNote X7.

In summary, important inclusion and exclusion criteria are as follows. We will include studies in which long-term care plans are explicitly indicated/described (e.g., moving into nursing home, formal or informal care provided at home, assisted living). We will include studies focusing on community dwelling individuals (18 years old and older). We will include studies investigating the link between sexual orientation and long-term care preferences, i.e., comparing gender and/or sexual minorities (e.g., homosexual vs. bisexual). Research focusing primarily on the following populations will be excluded: children, youths and research covering only one group (heterosexuals).

### 2.5. Data Extraction

From each eligible article, information about study design and methodology, participants, the definition and measurement of main variables (sexual orientation, long-term care preferences), demographics (age and sex) and baseline characteristics, statistical analysis, and key findings will be extracted. Two reviewers will extract or check received data (one researcher will extract data and the second one will verify the extracted data). Disagreements between the two reviewers (e.g., whether to include the study in the systematic review—more precisely, whether the studies should be excluded due to the sample size, the selection of study participants or due to outcome variables investigated) will be resolved through discussion to reach consensus or by inclusion of a third party. If clarification is required, study authors will be contacted for unreported data or additional details (using two contact attempts). The extracted data will be recorded in MS Excel spreadsheet and EndNote X7.

### 2.6. Quality Assessment

The study quality will be assessed using an appropriate quality assessment tool for observational cohort and cross-sectional studies such as the National Institutes of Health (NIH) Quality Assessment Tool for Observational Cohort and Cross-Sectional Studies [21]. According to the criteria of the NIH tool (e.g., participation rate, sample size justification, valid outcome measures) quality rating will be made. The studies will be classified in the following categories: good, fair, or poor. Moreover, we plan to assess the risk of bias (provided that obtained data allows it). We will examine the influence of low quality studies (quality assessment—“low”) on our results. In this case, only those studies with a “fair”- and “good”-quality assessment will be included in a further synthesis. We will assess whether these results differ from the results obtained from the synthesis including poor, fair and good studies [21]. Two reviewers will perform the quality assessment. If disagreements occur, they will be resolved through discussion or by inclusion of a third member of the research team. These results will be integrated in the systematic review.

### 2.7. Data Synthesis

The results will be synthesized in tables and summarized narratively [22]. All preferences for long-term care mentioned in the research (e.g., ageing at home, informal care, moving into a nursing home) will be listed. Results regarding long-term care preferences will be stratified by sexual orientation. If possible, results will be categorized according to age group. If data permit, a meta-analysis will be performed. After performing data synthesis, we will prepare the final systematic review following the Preferred Reporting Items for Systematic Reviews and Meta-Analysis (PRISMA) guidelines [22].

## 3. Discussion

As the proportion of individuals in old age is increasing, the long-term care preferences of ageing society are an important issue for both health policy and health care system. Within the ageing society, one should not neglect the large group of ageing sexual minorities. In our upcoming systematic review, we plan to synthesize the evidence about the long-term preferences with regard to sexual orientation.

It is expected that our review will provide evidence regarding the potential differences in long-term care preferences among LGBT community and heterosexuals. We hypothesize that LGBT individuals would prefer to be cared for at home compared to heterosexual individuals [23,24,25]. Due to the fear of discrimination and stigmatization, the preference to move into nursing homes may be lower among sexual minorities than among heterosexuals [18,26,27]. The review will further characterize and synthesize the preferences and the most favorable alternatives for long-term care that are considered by the LGBT community as well as heterosexuals, such as formal and informal care provided at home, moving into a nursing home, living will.

To our knowledge, this is the first systematic review that aims to describe the preferences for long-term care with regard to sexual orientation. Two recent systematic reviews considered some aspects of sexual minorities and long-term care [28,29]. Caceres et al. synthesized the perspectives of providers and LGBT individuals in long-term services and support in the United States [29]. Moreover, Mahieu et al. investigated the perceptions of community-dwelling LGBT individuals with regard to sexuality in residential aged care [28]. However, the important aspects of long-term care planning e.g., planning to move into nursing home, and the comparison of preferences between groups (e.g., heterosexuals vs. homosexuals) were not synthesized in these reviews. Therefore, our upcoming systematic review aims to close this gap in the literature.

In order to ensure high quality, we focused only on studies published in peer-reviewed, scientific journals in our systematic review. Thus, we cannot deny the possibility that some unpublished studies concerning the long-term care preferences of LGBT community will not be included in our systematic review.

Awareness about long-term preferences with regard to sexual orientation is important to improve our understanding of the potential differences in preferences among heterosexuals and sexual minorities. Moreover, this knowledge could contribute to a better understanding of the needs of ageing societies. More precisely, appropriate health care policies should incorporate the preferences of all groups within the society—including the under researched group of sexual and gender minorities.

## Figures and Tables

**Table 1:** Search strategy.

	Key Terms/String
(1) sexual orientation	(“lgbt *” OR “lgb” OR “glb” OR “homosexual *” OR “gay” OR “gays” OR “lesbian *” OR “bisexual *” OR “queer *” OR “sexual minorit *” OR “gender minorit *” OR “sexual orientation” OR “heterosexual *” OR “gsm” OR “sgm” OR “sexuality” OR “sexual expression” OR “gender identity“ OR “glbt *” OR “Lesbigay” OR “Men Who Have Sex With Men” OR “msm” OR “Non-Heterosexual” OR “Non-Heterosexuals” OR “sexual dissident *” OR “same-sex” OR “women who have sex with women” OR “wsw” OR “transgender” OR “transsexualism” OR “intersex” OR “gender non-conforming” OR “gender identi *” OR “ego-dystonic” OR “homosexism” OR “heterosexism” OR “homophobia” OR “stereotyping” OR “anti-Homosexual Bias” OR “anti-gay bias” OR “non-binary” OR “non binary” OR “same-sex”)
(2) long-term care	(“long-term care” OR “nursing home *” OR “NH” OR “residential care” OR “aged home *” OR “home care” OR “home health care” OR “home health” OR “assisted living” OR “LTC home *” OR “Old age home *” OR “institutionalisation” OR “institutionalization” OR “care home *” OR “care centre *” OR “care center *” OR “Home for the Aged” OR “Homes for the Aged” OR “Old Age Home” OR “Old Age Homes” OR “housing” OR “Intermediate Care Facilities” OR “Skilled Nursing Facilities” OR “Group Home *” OR “Residential Facilit *” OR “aged care” OR “rac” OR “senior hous *” OR “LTSS” OR “rehabilitation facility *” OR “subacute facility *” OR “adult day care” OR “entering” OR “entry” OR “end of life” OR “retirement care facilities” OR “formal assistance” OR “formal system*” OR “retirement facility *” OR “accommodation”)
(3) preferences	(“first choice” OR “expectation *” OR “need *” OR “attitude *” OR “willingness” OR “future plan *” OR “perception *” OR “option *” OR “planning” OR “plans” OR “intention *” OR “prefer *” OR “desire *” OR “wish *” OR “decision *” OR choice * OR priorit * OR “future needs” OR “housing” OR “motivation” OR “accommodation plan*” OR “concern *” OR “access *” OR “long term planning” OR “future living arrangement *”)
#1 AND #2 AND #3	

Note: The asterisk (*) is a truncation symbol.
